# Failure of Early Diagnosis of Infective Endocarditis in Japan—A Retrospective Descriptive Analysis

**DOI:** 10.1097/MD.0000000000000237

**Published:** 2014-12-12

**Authors:** Takahiko Fukuchi, Kentaro Iwata, Goh Ohji

**Affiliations:** From the Division of Infectious Diseases therapeutics, Department of Microbiology and Infectious Diseases, Kobe University Graduate School of Medicine, Kobe, Japan.

## Abstract

Infective endocarditis (IE) is a severe disease with high morbidity and mortality, and these can be exacerbated by delay in diagnosis. We investigated IE diagnosis in Japan with the emphasis on the delay in diagnosis and its cause and implications.

We conducted a retrospective study on 82 definite IE patients at Kobe University Hospital from April 1, 2008, through March 31, 2013. We reviewed charts of the patients for data such as causative pathogens, prescription of inappropriate antibiotic use prior to the diagnosis, existence of risk factors of IE, previous doctor's subspecialty, or duration until the diagnosis, with the primary outcome of 180-day mortality. We also qualitatively, as well as quantitatively, analyzed those cases with delay in diagnosis, and hypothesized its causes and implications.

Eighty-two patients were reviewed for this analysis. The average age was 61 ± 14.5-year-old. Fifty percent of patients had known underlying risk factors for IEs, such as prosthetic heart valve (10), valvular heart disease (21), congenital heart disease (3), or cardiomyopathy (2). The median days until the diagnosis was 14 days (range 2 days to 1 year). Sixty-five percent of patients received inappropriate antibiotic before the diagnosis (53). Forty percent of causative organisms were *Staphylococcus aureus* (MSSA 20, MRSA 13), 32% were viridans streptococci and *Streptococcus bovis*, 28% were others or unknown (CNS 5, *Corynebacterium* 3, *Cardiobacterium* 1, *Candida* 1). Subspecialties such as General Internal Medicine (15), and Orthopedics (13) were associated with delay in diagnosis. Ten patients (12%) died during follow up, and 8 of them had been received prior inappropriate antibiotics.

Significant delay in the diagnosis of IE was observed in Japan. Inappropriate antibiotics were prescribed frequently and may be associated with poor prognosis. Further improvement for earlier diagnosis of IE is needed.

## INTRODUCTION

Infective endocarditis (IE) is a severe disease with high morbidity and mortality in developed country (10–40%).^1,2^ Some studies reported that the mortality of IE in recent Japan ranges from 9% to 21.9%.^3–5^

Some argue that the accurate diagnosis and treatment of IE tends to be late in Japan, and it appears quite a number of patients suffer from serious complications, mainly due to delay in diagnosis and in transfer to appropriate referral hospitals.^6^

Japanese national insurance system enables Japanese citizen easily to seek medical care,^7^ although that might have led to easy visit to clinicians. Frequent visits may increase inappropriate prescription of antibiotics, that might lead to further delay in diagnosis of IE.

In this study, we hypothesized that there is significant delay in diagnosis of IE in Japan, and inappropriate use of antibiotics contributing to the delay. Therefore, we retrospectively investigated the time from the onset until the IE was thought as differential diagnosis, with the hypothesis that the duration being unacceptably long. Also we evaluated the relationship between inappropriate antibiotics, previous doctor's management, and prognosis, with their relatedness to the potential delay in the diagnosis of IE. We did analyze this both from quantitative and qualitative point of view, or descriptive way, to elucidate actual state of IE management in Japan.

## METHODS

This retrospective observational study was conducted on all patients diagnosed as IE at Kobe University Hospital, from April 1, 2008, through March 31, 2013. The hospital is a 928-bed teaching facility with division of cardiothoracic surgery and infectious disease. We enrolled 82 consecutive adult patients with definite IE according to modified Duke criteria.^8^

For each patient, data on age, sex, presence of co-morbidity (history of diabetes, cancer and hematological malignancy, cirrhosis, end-stage renal disease (ESRD) and hemodialysis (HD), immunosuppressive treatment, congenital immunodeficiency, and HIV/AIDS), risk factors of IE, causative pathogens, setting of infection whether it was acquired at community or healthcare, and requirement of surgical procedure, were collected according to existing guidelines and a previous study.^9^ Immunosuppressive treatment was defined as the administration of oral corticosteroids or immunosuppressive agents such as calcineurin inhibitors or antimetabolites. The risk factors for IE were defined as presence of prosthetic heart valve, valvular heart diseases, congenital heart diseases except for atrial septal defect, cardiomyopathy, past history of IE, and illicit intravenous drug users. Causative pathogens were defined as isolated organisms from blood cultures or surgical specimens. We acquired data from patient's medical records and the referral forms, the duration from the onset of initial symptoms to the physician's bringing up on the diagnostic hypothesis of IE.

We defined the duration as “delay” if the doctor was not aware of differential diagnosis of IE and did not bring IE to mind during the diagnostic work up to make our point clearer.

We also investigated previous doctors’ subspecialty since we hypothesized that it may be related to the doctor's ability to diagnose IE early. Concurrently, appropriateness of previous antibiotic use was also assessed. Any of oral antibiotics use after the onset of symptoms and antibiotics use under doses based on ESC (European Society of Cardiology) or AHA/IDSA (American Heart Association/Infectious Disease Society of America) guideline were defined as “inappropriate.”^10,11^

All statistical analyses were performed using STATA for Windows, release 13 (STATACorp LP, Texas). Univariate comparisons were made with Wilcoxon rank-sum test as appropriate.

Ethics committee at Kobe University Graduate School of Medicine approved this study.

## RESULTS

Eighty-two patients (53 males) with IE were reviewed for this analysis. The average age was 61 ± 14.5 years old (Table 1). Sixty-five percent of patients (53/82) received inappropriate antibiotic before the diagnosis. Most frequent causative organism was *S aureus* (40%, 33/82, with 20 MSSA and 13 MRSA), followed by viridans streptococci or *S bovis* (32%, 26/82), and others or unknown (28%, 21/82) (Table 2). The median duration from the onset until doctor's bringing up the diagnosis of IE was 14 days (ranging from 2 days to 1 year, Table 3). The subspecialties of physicians who failed to reach early notice of IE were General Internal Medicine (15, 18%), orthopedics (13, 16%), nephrologists (mainly engaged in HD) (4, 5%), although Cardiac surgeons (2, 2%) and Cardiologist (1, 1%) were more noticeable for early diagnosis of IE. Only 12% patients (10/82) were initially suspected of IE at the initial visit. Twelve percent of patients (10/82) died during follow up and 8 out of 10 had received prior inappropriate antibiotics (Table 4).

**TABLE 1:**
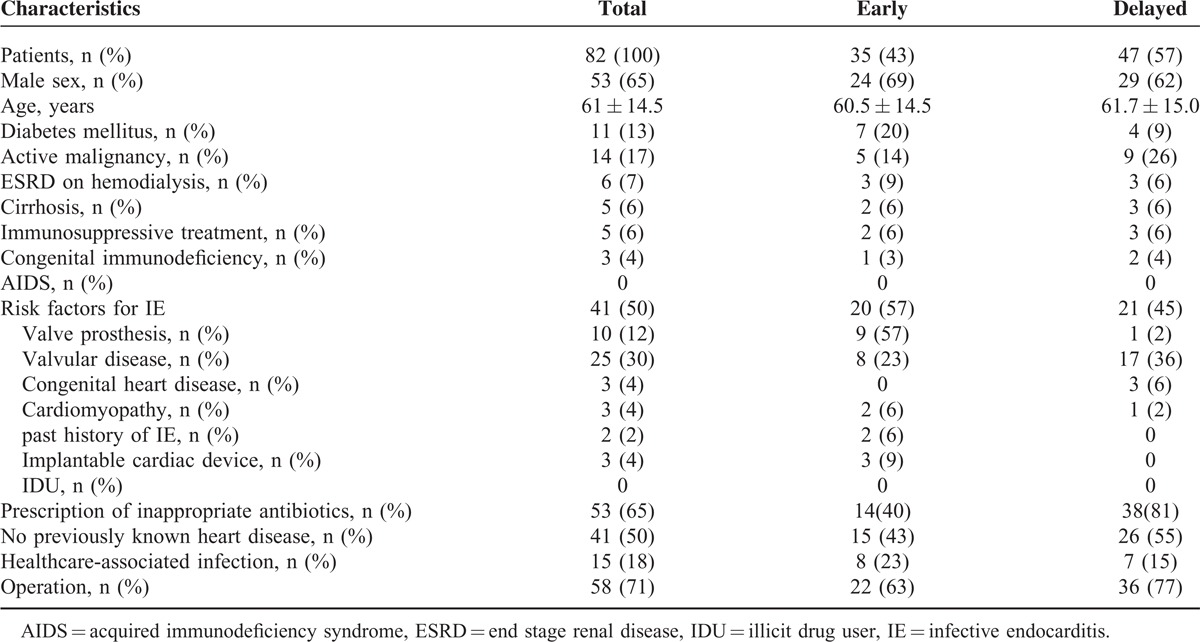
Population Characteristics

**TABLE 2:**
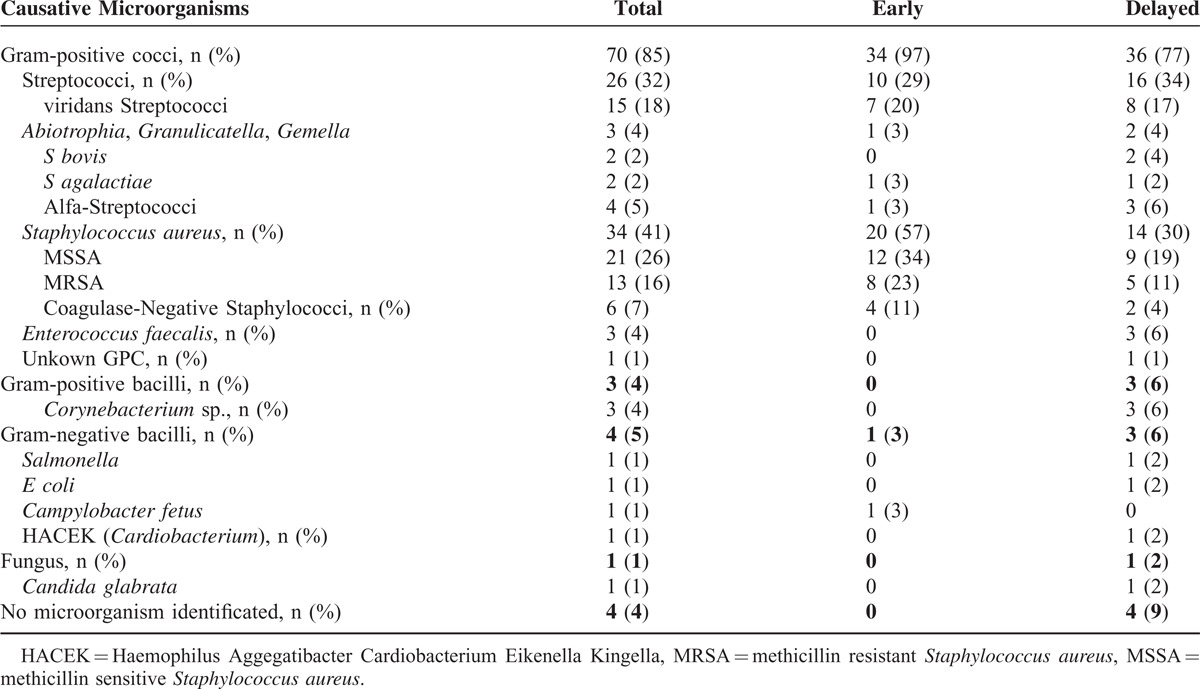
Characteristics of Causative Microorganisms

**TABLE 3:**
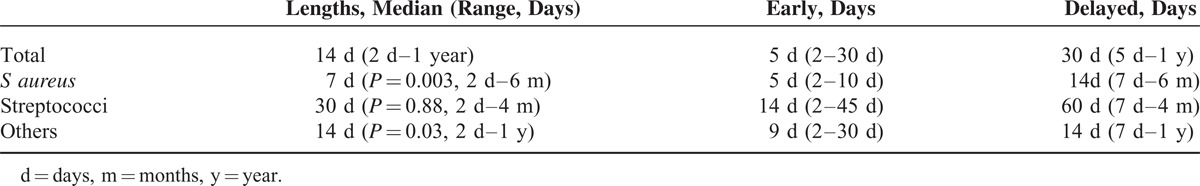
Duration for Bringing Up

**TABLE 4:**
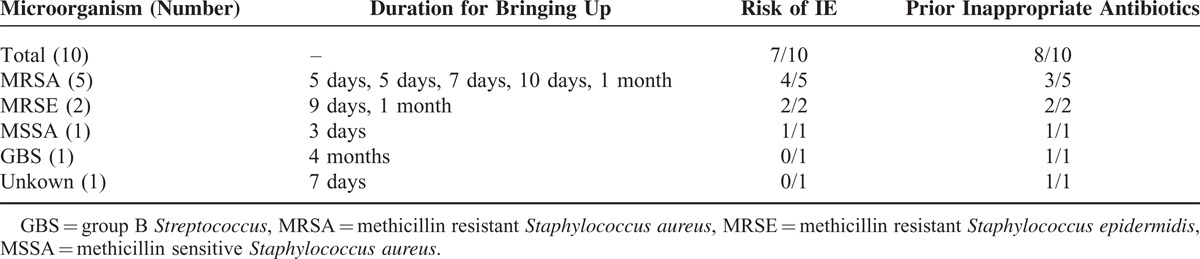
Characteristics of Patients Who Died

Unacceptable delay was observed in some cases, and we describe several of these in detail.

**FIGURE 1:**
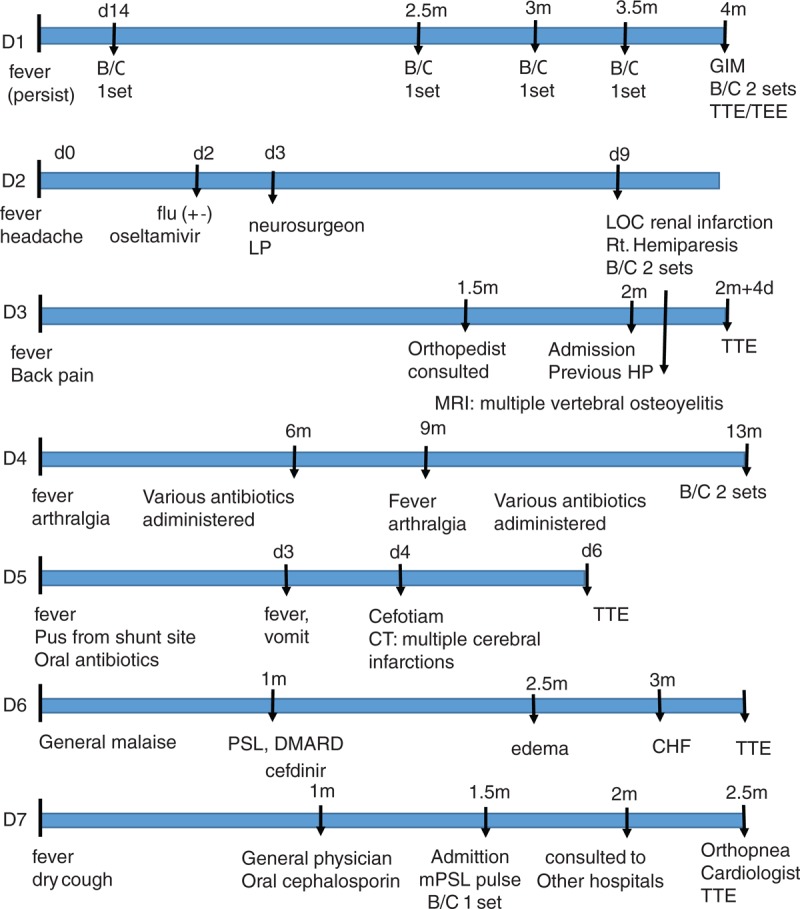
Timeline of patients. Examples of delayed diagnosed group.

Examples of delayed cases in Figure 1

1. D1: 65-year-old female who was diagnosed with ureter cancer developed intermittent fever since perioperative state, and subsequently was administered oral and intravenous antibiotics at each visit. Three times of blood culture (only 1 set of blood culture had been taken each through) were obtained and revealed *Corynebacterium striaatum* every time, but these results were considered as “contamination.” Four months since the onset, the urologist consulted a general internal medicine specialist, who advised to take 2 sets of blood cultures. *C. striaatum* was isolated from both sets.
2. D2: 19-year-old healthy female, who was known to have systolic murmur and otherwise healthy, developed fever and headache during a winter period. A general physician treated her as “influenza” with oseltamivir from the result of faintly positive rapid flu antigen, but symptoms persisted. The second doctor, a brain surgeon, treated her as “meningitis” with 4th generation cephalosporin and acyclovir because of slightly elevated CSF cell counts. On the 9th day she developed right hemiparesis, and transferred to our hospital. Two sets of blood cultures revealed MSSA.
3. D3: 56-year-old female, who had liver cirrhosis (Child B) due to HCV, developed persistent fever and back pain. The physician taking care of her cirrhosis and a private orthopedic doctor repeatedly administered oral and intravenous antibiotics for 2 months. MRI, which was taken by second orthopedist, revealed multiple vertebral osteomyelitis, and subsequently a cardiologist was consulted. Blood cultures revealed *Granulicatella elegans*.
4. D4: 61-year-old male who had ESRD on HD, developed fever and arthralgia. His nephrologist intermittently treated him as “intractable cellulitis” for 6 months with various antibiotics. Three months since his symptoms temporally diminished, fever and arthralgia re-developed. Various antibiotics, including anti-tuberculosis agents, were administered, but his symptoms persisted. After the consulting of general internal medicine physician, who recommended taking blood cultures, group B streptococcus (GBS) was isolated, and diagnosis of IE was given later.
5. D5: 61-year-old male, who had history of ESRD on HD and peripheral artery disease treated by bypass grafting, developed fever and inflammation at shunt site. His nephrologist drained that site, and administered oral third generation cephalosporin (cefcapene-pivoxil). His fever fluctuated, followed by vomiting 2 days later, so he was hospitalized to a hospital. Four days after admission his consciousness deteriorated despite IV cefotiam treatment. Brain CT was performed, which revealed multiple cerebral infarctions. On his 6th day, echocardiography was performed, which revealed mitral vegetation. He was transferred to this hospital, and MSSA was isolated from blood cultures.
6. D6: 78-year-old female, who had history of Parkinson disease, post-operative state of cerebral aneurysm, mitral regurgitation, and aortic regurgitation, presented with general malaise and loss of body weight. A private orthopedist diagnosed her as “rheumatoid arthritis” because her serum anti-nuclear antigen (ANA) and rheumatoid factor (RF) were positive 1 month after the symptoms, and administered prednisolone and actarit, a kind of DMARDs (disease modifying antirheumatic drugs), which was not approved except for Japan. Around the same time, a private general physician prescribed oral third generation cephalosporin (cefdinir) for her vague general symptoms. She developed lower limb edema 1 month later, so she was admitted to a hospital. At that hospital, she was taken 3 sets of blood cultures, which had remained negative. Echocardiography was performed, because congestive heart failure developed, which revealed mitral vegetation. After transferring to our hospital and cardiac operation, *Cardiobacterium hominis* was isolated from blood culture taken at the previous hospital.
7. D7: 32-year-old healthy male developed fever, dry cough, and gradually increasing dyspnea. He was admitted to a hospital 1 month after the onset of symptoms. He was diagnosed as “interstitial pneumonitis” because of his ground glass opacity on chest CT, and administered methyl-prednisolone pulse therapy (1 g for 3 days), followed by oral prednisolone, which could decrease his symptoms in short term. Although his blood culture revealed viridans streptococci, it was considered as contamination. Tapering his prednisolone, his symptoms relapsed and deteriorated, so he was consulted to other 2 hospitals (and seen by a pulmonary medicine specialist and a hematologist) 2 months later. None of these mentioned anything about IE. Orthopnea occurred, and he visited a private general cardiologist 2.5 months after the onset. Acute mitral regurgitation was revealed by echocardiography, then he was admitted our hospital. *Streptococcus parasanguis* was isolated from his blood cultures.

**FIGURE 2:**
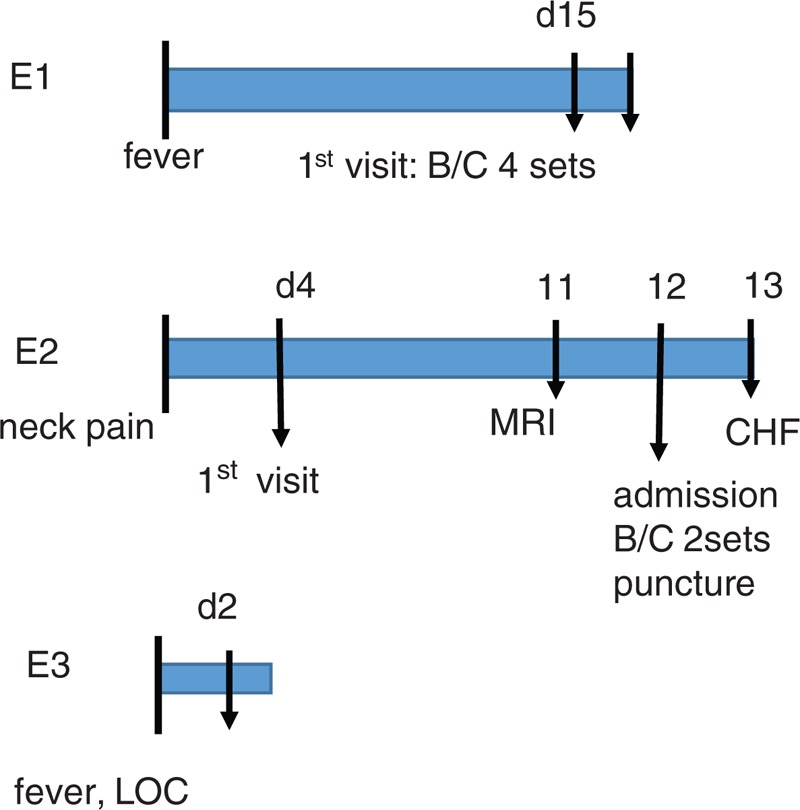
Timeline of patients. Examples of Early diagnosed group.

Exemplary early diagnoses occurred some times, and some cases were shown below (Figure 2).

1. E1: 68-year-old male with past history of medically treated IE visited a hospital with 2 weeks of fever. The physician immediately took 4 sets of blood cultures, and Streptococcus salivarius was recovered.
2. E2: 64-year-old male who was diagnosed as pyogenic diskitis by imaging studies. Needle aspiration of the lesion, as well as blood cultures revealed GBS.
3. E3: 76-year-old male who had past history of ESRD on HD developed fever and loss of consciousness. On the next visit to HD clinic, the nephrologist took 2 sets of blood cultures as per an advice from an intensivist. MSSA was isolated.

## DISCUSSION

We presented the current status of IE diagnosis in Japan, both qualitatively and quantitatively. The status is far from ideal and we observed unacceptable delay in diagnosis of IE and erratic use of antibiotics, together with inappropriate use and misinterpretation of blood cultures.

Even though half the patients had underlying risk factors associated with IE, only 12% of patients were suspected of possible IE at the first visit. There was lack of rational assessment and appropriate provisional diagnosis. Both oral and intravenous antibiotics were provided without appropriate diagnostic work up.

The rate of prior antibiotic prescription reached to 65%, which was higher than the countries (48–54%).^12,13^ Another Japanese study revealed 59% (92/155) of patients with IE had received prior antibiotics.^4^ Even huge steroid therapy was sometimes provided, which might worsen the prognosis of the patients. The diagnosis of IE was not brought up frequently, particularly among those who are not cardiologists or cardiac surgeons, suggesting indifference to diseases other than one occurring to the organ outside their specialties. This indifference was referred to “Tako Tsubo,” Japanese term for octopus pot, as many Japanese specialists enter into a pot of knowledge and they do not go outside, with indifference to other area of specialties.^14^ Despite persistent fever, ambiguous antibiotics therapy was continued without consultation with infectious diseases specialists. They do not seek help from other specialists often as Japanese physicians traditionally treated patients inside the division, without consulting other department.

Unnecessary antibiotic might be associated with very good accessibility to medical care in Japan.^7^ Patients expect doctors prescribe “something” and feel unfair if they were not given medications after the visit. Private doctors might be forced to prescribe antibiotics for fear of losing their patients, but this behavior was actually disadvantageous to their patients.

We hypothesized the relationship between inappropriate antibiotics prescription and higher mortality. Then, the prescription rate in died patients tend higher rate (80%), however, there was not any significant difference (*P* = 0.17). This may be caused by scanty power and less overall mortality rate. Further studies are needed to confirm this hypothesis.

Some other factors may be associated with the delay in diagnosis of IE. First, there was no reimbursement for 2 sets of blood cultures until April 2014 at Japanese National Health Insurance, this fact might have reduced the incentive of taking these for diagnosis of IE or any kind of bacteremia. Positive result of only one set of blood culture easily misleads doctors to interpret results as contamination (D1, D7). Second, Japanese doctors have tended to consider IE as an uncommon disease, according to a Japanese study.^15^ Education about IE and importance of blood cultures for doctors may be helpful.

The duration till bringing up of streptococcal IE (median 14 days, range 2 days–4 months) and staphylococcal IE (median 7 days, range 2 days–6 months) were shorter than duration for diagnosis of researches in era that echocardiography was widely available (15 ± 19 days),^9^ (68 ± 17 days).^16^ However, the duration of the most elongated case reached 4 months, 6 months, and 1 year, which was caused by intermittent oral or intravenous antibiotics without assessment of IE (D1–D7).

This tendency was particularly conspicuous among General Internal Medicine (GIM) physicians and Orthopedic surgeons. A Japanese nationwide study about vertebral osteomyelitis revealed that 2% (145/7118) of vertebral osteomyelitis episodes supervened with IE.^17^ In our series, the Orthopedists often did not notice of IE when they treated vertebral osteomyelitis. Figure 1 shows several examples of timeline of early (E1–E3) and delayed (D1–D7) diagnosed IE. The previous doctor's specialties were urologist (D1), GIM and neurosurgeon (D2), GIM and orthopedist (D3), and nephrologist (D4, D5), GIM and orthopedist (D6), pulmonologist (D7).

The cause of death was most associated with causative microorganisms, such as *S aureus*, although there was not any significant difference (*S aureus* 18% vs others 8%; *P* = 0.65). The patients who died in staphylococcal endocarditis were mostly caused by associated comorbidities (advanced cardiomyopathy, multiple organ failure, not want to invasive procedure, brainstem infarction, and multiple cerebral infarction).

In addition, the duration for diagnosis of IE of *S aureus* differed from earlier in nosocomial infection, especially in the institute where Infectious Disease physician and Cardiologist were working full-time.

There are several limitations in our study. First, the patients were retrospectively evaluated, there may be some confounding factors. Second, the rate of surgical procedure among our patients was very high, suggesting the characteristics of our patients might not reflect the same population of IE in Japan, because Kobe university hospital is well known for its excellence in cardiovascular division. Third, though we defined “delay” as over 3 times of visit after the development of the patient's symptom on outpatient clinic setting, or over 3 days of hospital stay on hospitalization setting, “delay” was arbitrary assumption.

## CONCLUSION

We described the demographics of IE, which were treated in a Japanese university hospital. Inappropriate antibiotic administration may have lengthened the duration for diagnosis of IE. Further improvement for earlier diagnosis will be required. Everlasting educational activities about IE to many people, ranging from medical students to private physicians are needed.
